# Attendance at antenatal clinics in inner-city Johannesburg, South Africa and its associations with birth outcomes: analysis of data from birth registers at three facilities

**DOI:** 10.1186/s12889-017-4347-z

**Published:** 2017-07-04

**Authors:** Siphamandla Gumede, Vivian Black, Nicolette Naidoo, Matthew F. Chersich

**Affiliations:** 1 Wits Reproductive Health and HIV Institute (WRHI), Faculty of Health Sciences, University of the Witwatersrand, Johannesburg, South Africa; 2 Clinical Microbiology and Infectious Diseases, Faculty of Health Sciences, University of the Witwatersrand, Johannesburg, South Africa

**Keywords:** Antenatal attendance urban health, Maternal health, South Africa, Birth outcomes, PMTCT, Adolescents, Stillbirths

## Abstract

**Background:**

Antenatal care (ANC) clinics serve as key gateways to screening and treatment interventions that improve pregnancy outcomes, and are especially important for HIV-infected women. By disaggregating data on access to ANC, we aimed to identify variation in ANC attendance by level of care and across vulnerable groups in inner-city Johannesburg, and document the impact of non-attendance on birth outcomes.

**Methods:**

This record review of routine health service data involved manual extraction of 2 years of data from birth registers at a primary-, secondary- and tertiary-level facility within inner-city Johannesburg. Information was gathered on ANC attendance, HIV testing and status, pregnancy duration, delivery mode and birth outcomes. Women with an unknown attendance status were considered as not having attended clinic, but effects of this assumption were tested in sensitivity analyses. Multiple logistic regression was used to identify associations between ANC attendance and birth outcomes.

**Results:**

Of 31,179 women who delivered, 88.7% (27,651) had attended ANC (95% CI = 88.3–89.0). Attendance was only 77% at primary care (5813/7543), compared to 89% at secondary (3661/4113) and 93% at tertiary level (18,177/19,523). Adolescents had lower ANC attendance than adults (85%, 1951/2295 versus 89%, 22,039/24,771). Only 37% of women not attending ANC had an HIV test (1308/3528), compared with 93% of ANC attenders (25,756/27,651). Caesarean section rates were considerably higher in women who had attended ANC (40%, 10,866/27,344) than non-attenders (13%, 422/3360). Compared to those who had attended ANC, non-attenders were 1.6 fold more likely to have a preterm delivery (95% CI adjusted odds ratio [aOR] = 1.4–1.8) and 1.4 fold more likely to have a stillbirth (aOR 95% CI = 1.1–1.9). Similar results were seen in analyses where missing data on ANC attendance was classified in different ways.

**Conclusion:**

Inner-city Johannesburg has an almost 5% lower ANC attendance rate than national levels. Attendance is particularly concerning in the primary care clinic that serves a predominantly migrant population. Adolescents had especially low rates, perhaps owing to stigma when seeking care. Interventions to raise ANC attendance, especially among adolescents, may help improve birth outcomes and HIV testing rates, bringing the country closer to achieving maternal and child health targets and eliminating HIV in children.

**Electronic supplementary material:**

The online version of this article (doi:10.1186/s12889-017-4347-z) contains supplementary material, which is available to authorized users.

## Background

The Sustainable Development Goal (SDG) that speaks directly to health (Goal 3) makes commitments to reduce the maternal mortality ratio to under 70 deaths per 100,000 live births, prevent deaths of newborns and children under 5, and reduce mortality related to Human Immunodeficiency Virus (HIV) and other communicable diseases [1]. High coverage of antenatal care (ANC) and of interventions for preventing mother-to-child transmission (PMTCT) [2] of HIV will be important for achieving these commitments. The SDGs place much emphasis on the need for reducing inequalities, and that targets will only be considered achieved if they are have been met for all relevant income and social groups [3]. Identifying groups that require additional attention and the monitoring of progress thus requires disaggregated data, including by place of residence and migrant status.

Rates of ANC attendance, however, remain suboptimal in many low- and middle-income countries (LMICs) [4, 5]. Within the southern and eastern regions of Africa, coverage ranges from 60% in Lesotho to 95% in Rwanda, with most countries around the 80% mark [6]. South African ANC coverage was 90% in a national household survey in 2012 [7] and 93% in 2014, according to the district health information system [8].

Population and community-based studies in several countries, including Nepal, South Africa and Zambia [9–11], have noted the consequences of ANC non-attendance. Similarly, in South Africa, successive national audits of maternal deaths have documented the large number of deaths that may be attributed, at least in part, to poor attendance at ANC [12–14].

In South Africa, while there are data on antenatal attendance at a district level [15], more detailed information is needed on ANC attendance and its impacts on vulnerable inner-city populations. This is especially true for migrant populations who make up a large proportion of residents in many areas and often live in informal over-crowded housing [16]. A previous study in the site showed that compared to local women, those who were not of South African decent often experienced difficulties in obtaining an appointment for the ANC [17]. The reasons given by health workers for delaying their appointment were seldom substantiated and not in line with government antenatal care guidelines. Therefore, it is important to quantify the impact of inadequate access to ANC on birth outcomes, particularly in a setting with a highly transient population.

Addressing service gaps is particularly important for achieving the goal of eliminating HIV infections in children, as these urban areas have amongst the highest prevalence of HIV in the world and antenatal clinics are the principal gateways to PMTCT services [18–20]. In this record review of routine health service data, we thus aimed to determine the proportion of women attending ANC in inner-city Johannesburg, to consider how this varies by level of care and to identify sub-groups with especially low utilisation. Associations between ANC attendance and birth outcomes were also assessed.

## Methods

The study included the public health facilities where all public-sector births within the inner-city, region F of Johannesburg take place. Specifically, these were: Hillbrow Community Health Centre (HCHC, a primary health clinic), South Rand Hospital (SRH, a secondary-level district hospital and Charlotte Maxeke Johannesburg Academic Hospital (CMJAH, a tertiary-level facility). Private sector hospitals were not included as we believed that it would be difficult to access data from patients at those facilities and they make up a relatively small proportion of all deliveries in the region. We manually extracted data from labour ward registers of all births in 2008 and 2009 at HCHC and SRH. Data were also extracted from birth registers at CMJAH for the years 2011 and 2012. All births are captured in these registers, as well as information on deliveries that occur before the mother and infant arrive at the facility. The registers collate pregnancy, intrapartum and birth outcomes data, and are a rich source of information.

### Description of study setting

A protocol describing the care processes in the region states at which level of care the different patient groups are to receive treatment. In HCHC, nurses and midwives provide care for women with uncomplicated pregnancies. SRH, the secondary level of care is managed by midwives and medical officers who treat women with minor medical complications. At CMJAH all women are assessed by a doctor and there are medical specialists available for more complicated patients. Women are considered to have a complicated pregnancy if they were unwell in a previous pregnancy, or have, for example, co-morbid medical illnesses, multiple gestations or suspected congenital abnormalities. In HCHC and SRH, women do not require a referral letter and can present directly for services when pregnant. CMJAH only provides ANC for women with pregnancy complications and then refers healthy women to lower levels of care. Caesarean sections are only performed at the secondary- and tertiary-level facilities. There are no user fees for HIV, ANC or childbirth services. HIV testing, PMTCT and antiretroviral treatment are available at all facilities.

It is important to note that the study setting, region F (inner-city), has several distinctive features which influence the uptake of health services and programme implementation. The inner-city is one of seven regions within the district called City of Johannesburg. In turn, the City of Johannesburg is one of five districts of the Gauteng Province, the economic hub of the country. The inner-city is densely populated, consisting of the flatland areas of Hillbrow and Berea, as well as the Johannesburg Central Business District. It is estimated that the region contains about 15% of the 4 million people who live in the city of Johannesburg [21]. However, it is likely that this figure is well above 15% as the majority of the large transient population in the inner-city area are not included in those estimates. The inner-city is a uniquely complex and dynamic environment that has undergone major demographic, social and economic shifts over the last few decades. Many of its inhabitants are immigrants, both South Africans from other provinces of the country as well as foreigners. About a quarter of adults in the area are unemployed [21]. The inner-city houses several large taxi hubs and 800,000 commuters are said to pass through the city daily. Informal trading is the dominant economic activity. Unfortunately, the area is plagued with high levels of crime and drug dealing [22]. Not surprisingly, the area has a high burden of HIV: the antenatal HIV prevalence in Gauteng Province as a whole was 29.9% in the most recent national survey (2012), with a similar level in the City of Johannesburg (29.6%). Corresponding figures for Herpes Simplex Virus type-2 were 58.4% and 58.5% [23].

### Data management and study variables

The following variables were manually extracted from the labour ward registers into a Microsoft Excel database: ANC attendance (having attended at least one ANC visit), maternal age (not available at SRH), gravidity and parity (only available at CMJAH), HIV testing uptake and status, mode of delivery, infant gender, gestation at childbirth and stillbirths. Patient names or other identifiers were not collected.

Adolescents were divided into younger and older groups (10–16 years and 17–19 years), which correspond broadly to the ages of those in primary or secondary school (10–16 years), and those in the final years of school or post-school (17–19 years). Adults were groups into 5-year age bands. As about 4% of data were missing on whether women had attended ANC, we performed a sensitivity analysis in which we reclassified the missing data in one of three ways. Firstly, in a worst case scenario, women whose attendance status was unknown were classified as not having attended ANC. In the second scenario, a ‘missing excluded’ analysis, we excluded all women where it was unknown whether or not they had attended ANC. The third scenario, the best case, classifies women with unknown attendance as having attended ANC. Findings across the three scenarios were compared for effect size and direction. The associations reported in the paper are drawn primarily from the worst case scenario, as we considered it most likely that where attendance was unknown, patients had not actually attended care. Also, many of the figures in the other two scenarios were implausible. Figures for HCHC were unchanged across the scenarios as data were not missing on ANC attendance at that site.

Newborns were classified as preterm (under 37 weeks gestation), term (37 to 41 weeks) and post-term (42 or more weeks). Stillbirths included some deaths that occurred shortly after birth as the birth records did not differentiate between those deaths and actual stillbirths.

### Data analysis

Data were recoded and analysed using STATA version 12 (STATA Corporation, College Station, Texas, USA). Chi-square tests were used to detect associations between categorical variables and Wilcoxon Rank sum tests were used for analysis of continuous variables. Pearson Chi-square test for trend assessed associations between ordinal exposure categories and binary outcomes. Multiple logistic regression models examined whether ANC attendance was associated with two dependent variables assessing service access (HIV testing and Caesarean section delivery) and two birth outcomes (preterm births and stillbirth). These models included the variable site, and other potential confounding variables associated with the outcome in univariate analysis or in analysis stratified by site (*P* < 0.10). The exposure variable antenatal attendance was included in the multivariate model assessing factors associated with the outcome preterm delivery (attendance was associated with preterm delivery in all three facilities, even though not associated with the outcome when data from all sites were pooled). For similar reasons, the exposure variable delivery mode was included in the multivariate model assessing factors associated with the outcome stillbirths. Potential confounders varied by outcome variable, with, for example, infant sex not included in the outcome HIV testing, as there was no plausible causal pathway between these variables. Infant sex, however, and HIV status were considered potential confounders for the remaining three models.

## Results

### Characteristics of women and birth outcomes

As shown in Table 1, of all 31,179 births reviewed, 24% took place at HCHC (7543), 13% at SRH (4113) and 63% at CMJAH (19,523). The median age of women at HCHC was 25 years (IQR = 21–28), lower than the median 27 years at CMJAH (IQR = 23–31). Of all deliveries at the primary and tertiary facility (27,066), 9% were in adolescents (2295); with a higher proportion at HCHC (11%, 819) than that at CMJAH (8%, 1476). There were considerably more women aged above 30 years at CMJAH than at HCHC (35%, *n* = 6828 versus 21%, *n* = 1555).

**Table 1:** Maternal characteristics, antenatal clinic attendance and birth outcomes at a primary, secondary and tertiary level facility in inner-city Johannesburg, South Africa

Variable	Primary care clinic (HCHC)	Secondary level hospital (SRH)	Tertiary hospital (CMJAH)	*P*
	% (n/N)	% (n/N)	% (n/N)	
Maternal age (years)				
10–16	1.2 (88/7543)	-	1.3 (252/19,523)	<0.001
17–19	9.7 (731/7543)		6.3 (1224/19,523)	
20–24	38.3 (2894/7543)		26.4 (5154/19,523)	
25–29	30.2 (2275/7543)		31.1 (6065/19,523)	
30–34	14.3 (1082/7543)		20.7 (4051/19,523)	
≥ 35	6.3 (473/7543)		14.2 (2777/19,523)	
Parity				
1	-	-	55.9 (6461/11,554)	-
2–4			40.0 (4616/11,554)	
≥ 5			4.1 (477/11,554)	
Gravidity				
1	-	-	30.3 (5708/19,382)	-
2–4			54.3 (10,664/19,382)	
≥ 5			15.4 (3010/19,382)	
Attendance at ANC^b^				
Worst case scenario^a^	77.1 (5813/7543)	89.0 (3661/4113)	93.1 (18,177/19,523)	<0.001
Missing are excluded^b^	77.1 (5813/7541)	99.1 (3661/3693)	97.0 (18,177/18,745)	<0.001
Best case scenario^c^	77.1 (5813/7543	99.2 (4081/4113)	97.1 (18,955/19,523)	<0.001
Had an HIV test	68.5 (5167/7543)	88.0 (3621/4113)	93.6 (18,276/19,523)	<0.001
HIV positive^d^	36.7 (1897/5167)	23.9 (867/3621)	29.8 (5452/18,276)	<0.001
Caesarean section rate	NA	24.0 (984/4102)	54.1 (10,304/19,048)	<0.001
Infant sex				
Female	51.0 (3833/7520)	49.4 (2031/4110)	48.9 (8636/17,653)	0.01
Male	49.0 (3687/7520)	50.6 (2079/4110)	51.1% (9017/17,653)	
Gestation at birth				
Preterm	0.3 (26/7543)	1.1 (47/4113)	26.9 (5125/19,022)	<0.001
Term	99.7 (7517/7543)	98.7 (4058/4113)	68.3 (12,989/19,022)	
Post-term	0 (0/7543)	0.2 (8/4113)	4.8 (908/19,022)	
Stillbirth^e^ (n deaths per 1000 live births)	4.3/1000 (32/7510)	3.9/1000 (16/4093)	28.0/1000 (487/17,401)	<0.001

*NA* not applicable as caesarean sections not done at HCHC.

–Data not collected in labour ward registers

^a^Worst case scenario: number of women who attended ANC among all women (women with unknown attendance classified as not having attended ANC)

^b^Missing excluded: number of women known to have attended ANC, among those whose ANC attendance status was known (women with unknown ANC attendance are excluded)

^c^Best case scenario: women with unknown attendance classified as having attended ANC

^d^HIV status among women with a known status

^e^Includes death shortly after birth

In total, 87% of women had a known HIV status (27,064). Testing levels were highest in CMJAH (94%), but, of concern, considerably lower in SRH (88%) and HCHC (69%). In multivariate analysis, after adjusting for ANC attendance, HIV testing was 5.4 fold higher in CMJAH than HCHC (95% CI adjusted odds ratio [aOR] = 4.9–5.9; Table 2).

**Table 2:** Associations between antenatal attendance, and access to other services and birth outcomes in worst case scenario (women with unknown attendance classified as not having attended ANC)

Variable	Univariate odds ratio	*P*	Multivariate odds ratio	*P*
Had an HIV test				
Attended ANC				
Yes	1.0	<0.001	1.0	<0.001
No	0.04 (0.04–0.05)		0.05 (0.05–0.06)	
Site				
Primary level	1.0		1.0	
Secondary level	3.38 (3.04–3.76)	<0.001	3.01 (2.66–3.41)	<0.001
Tertiary level	6.74 (6.25–7.27)	<0.001	5.36 (4.92–5.85)	<0.001
Had a Caesarean section				
Attended ANC				
Yes	1.0	<0.001	1.0	<0.001
No	0.34 (0.31–0.38)		0.39 (0.35–0.44)	
Site				
Secondary level	1.0	<0.001	1.0	<0.001
Tertiary level	3.75 (3.47–4.05)		3.35 (3.10–3.63)	
HIV status				
Negative	1.0		1.0	
Positive	1.06 (1.00–1.13)	0.04	1.0 (0.94–1.06)	0.98
Unknown	0.50 (0.45–0.56)	<0.001	0.64 (0.57–0.72)	<0.001
Gestation at childbirth				
Term or post-term	1.0	<0.001	1.0	<0.001
Preterm	1.46 (1.37–1.56)		1.24 (1.17–1.33)	
Preterm birth				
Attended ANC				
Yes	1.0	0.77	1.0	<0.001
No	1.01 (0.92–1.11)		1.59 (1.40–1.79)	
Site				
Primary level	1.0	<0.001	1.0	<0.001
Secondary level	3.34 (2.07–5.40)		5.41 (3.34–8.78)	
Tertiary level	106.6 (72.5–156.9)	<0.001	216.9 (146.1–322.1)	<0.001
Infant sex				
Male	1.0	0.55	-	-
Female	0.98 (0.92–1.05)			
HIV status				
Negative	1.0	<0.001	1.0	<0.001
Positive	1.25 (1.16–1.33)		1.33 (1.23–1.43)	
Unknown	1.13 (1.03–1.24)	<0.001	4.01 (3.53–4.55)	<0.001
Stillbirth^a^				
Attended ANC				
Yes	1.0	<0.001	1.0	0.02
No	2.0 (1.6–2.4)		1.40 (1.06–1.85)	
Site				
Primary level	1.0	0.78	1.0	0.08
Secondary level	0.92 (0.50–1.67)		1.73 (0.93–3.19)	
Tertiary level	6.57 (4.59–9.40)	<0.001	16.6 (11.2–24.7)	<0.001
HIV status				
Negative	1.0		1.0	
Positive	1.12 (0.90–1.38)	0.31	1.09 (0.87–1.36)	0.46
Unknown	2.38 (1.93–2.93)	<0.001	4.24 (3.29–5.48)	<0.001
Delivery mode				
Vaginal delivery	1.0	0.14	1.0	<0.001
Caesarean section	0.87 (0.72–1.05)		0.5 (0.4–0.6)	
Infant sex				
Male	1.0	0.004	1.0	0.004
Female	0.77 (0.65–0.92)		0.76 (0.64–0.92)	

Caesarean sections are not done at HCHC. No births considered post-term. Worst case scenario is the number of women who attended ANC among all women (women with unknown attendance classified as not having attended ANC)

^a^Includes death shortly after birth

Of those who had a test, 30% were HIV positive (8216). These 8216 women would have required PMTCT services (this reflects the burden of care relating to HIV in these three facilities). Considerable differences were noted in HIV prevalence between sites: it was highest in HCHC (37%, 1897), lower in CMJAH (30%, 5452) and lowest in SRH (24%, 867). At SRH and CMJAH, the prevalence of HIV was similar among women who had and had no attended ANC. At HCHC, however, HIV prevalence was 65% in the 144 non-attenders who had been tested, almost 20% higher than the prevalence in attenders.

Overall, for the sub-district, 36% of deliveries were by Caesarean section (11,288/31,179), with levels reaching 54% in CMJAH (10,304/19,048). Caesarean section was considerably less common in women with an unknown HIV status, even after controlling for other variables (Table 2). The odds of having a Caesarean section were 1.2 fold higher in preterm than term gestations, and similar in women with or without HIV infection. At CMJAH, 52% of women with a live newborn were delivered by Caesarean section (9027/17,270), compared to only 33.9% of stillbirth deliveries (156/460). Very negligible differences in infant gender were noted between facilities, with slightly more males delivered in higher levels of care.

In total, 17% of all newborns were classified as preterm babies (5198/30,678). Almost all deliveries categorised as preterm took place at CMJAH (99%, 5125/5198), where 27% of newborns were preterm. Rates of preterm delivery were 1.3 fold higher in HIV positive than negative women (aOR = 1.2–1.4), and 4 fold higher in women with an unknown HIV status, even after controlling for ANC attendance (Table 2).

Overall, the stillbirth rate was 18 per 1000 live births (535/29,004). Stillbirth rates were several fold higher in CMJAH than in HCHC and SRH (28/1000 in CMJAH versus about 4/1000 for HCHC and SRH). Independent of ANC attendance status and other potential confounders, having an unknown HIV status was associated with stillbirths (aOR = 4.2, 95% CI = 3.3–5.5). Stillbirths were also more common among female than male infants (aOR = 0.76, 95% CI = 0.64–0.92).

### Overall antenatal attendance and variations among sub-groups

ANC attendance data were available for all women at HCHC, 90% of women at SRH (3693) and 96% at CMJAH (18,745). Overall, utilisation of ANC was 88.7% in the worst case scenario, which classified those with missing data as not having attended ANC (95% CI = 88.3–89.0; 27,651/31,179). CMJAH had the highest attendance (93%, 18,177/19,523) and HCHC the lowest (77%, 5813/7543; Table 3). If attendance estimates exclude women where ANC attendance was unknown (scenario with missing excluded), then ANC utilisation was 99% in SRH (3661/3693), 97% at CMJAH (18,177/18,745) and 77% at HCHC (5813/7541; Additional file 1: Table S1). The percentages in the best case scenario (missing classified as having attended) were very similar to the scenario where missing data were excluded (Additional file 2: Table S2). Associations detected between the characteristics of women and attendance were similar in all scenarios.

**Table 3:** Proportion of women attending antenatal care in each facility, by maternal characteristics and birth outcomes in worst case scenario (women with unknown attendance classified as not having attended ANC)

Variable	Primary care clinic	*P*	Secondary level hospital	*P*	Tertiary hospital	*P*
	% attended (n/N)		% attended (n/N)		% attended (n/N)	
ANC attendance for facility	77.1 (5813/7543)		89.0 (3661/4113)		93.1 (18,177/19,523)	
Maternal age (years)						
10–16	75.0 (66/88)	0.01^$^	-	-	91.0 (229/252)	<0.001^$^
17–19	74.1 (542/731)				91.0 (1114/1224)	
20–24	75.9 (2197/2894)				91.7 (4726/5154)	
25–29	79.0 (1797/2275)				93.6 (5674/6065)	
30–34	78.0 (844/1082)				94.1 (3814/4051)	
≥ 35	77.6 (367/473)				94.3 (2620/2777)	
Parity^b^						
1	-	-	-	-	93.4 (6046/6461)	0.85^$^
2–4					93.4 (4642/4966)	
≥ 5					93.9 (448/477)	
Gravidity^b^						
1	-	-	-	-	92.0 (5252/5708)	<0.001^$^
2–4					93.4 (11,761/12,581)	
≥ 5					94.3 (1040/1093)	
Had HIV test						
Yes	97.2 (5023/5167)	<0.001	92.6 (3353/3621)	<0.001	95.1 (17,380/18,276)	<0.001
No	33.3 (790/2376)		62.6 (308/492)		63.9 (797/1247)	
HIV status^a^						
Positive	95.0 (1803/1897)	<0.001	92.0 (798/867)	0.47	95.0 (5180/5452)	0.72
Negative	98.5 (3220/3270)		92.8 (2555/2754)		95.1 (12,200/12,824)	
Delivery mode						
Vaginal delivery	77.1 (5813/7543)	-	87.6 (2741/3129)	<0.001	90.6 (7924/8744)	<0.001
Caesarean section	NA		93.5 (920/984)		96.5 (9946/10,304)	
Infant sex						
Female	77.7 (2976/3833)	0.28	88.4 (1795/2031)	0.19	94.0 (8115/8636)	0.24
Male	76.6 (2824/3687)		89.7 (1864/2079)		93.5 (8434/9017)	
Gestation at birth						
Preterm	57.7 (15/26)	0.02	80.9 (38/47)	0.20	88.8 (4551/5125)	<0.001
Term	77.1 (5798/7517)		89.1 (3616//4058)		94.8 (12,318/12,989)	
Post-term	-		87.5 (7/8)		95.4 (866/908)	
Infant status at birth						
Alive	77.2 (5799/7510)	<0.001	89.1 (3647/4093)	0.32	94.1 (16,367/17,401)	<0.001
Stillbirth^b^	40.6 (13/32)		81.3 (13/16)		83.2 (405/487)	

At HCHC, no births were considered post-term. Worst case scenario is the number of women who attended ANC among all women

*NA* not applicable as caesarean sections not done at HCHC

–Data not collected in labour ward registers

^$^Chi-square test for trend

^a^HIV status among women with a known status

^b^Includes death shortly after birth.

In CMJAH and HCHC, ANC attendance increased step-wise with age of the woman, though it declined from 35 years upwards at HCHC. In the worst case scenario, adolescents were 1.4 fold more likely not to have attended ANC than adults (95% CI OR = 1.3–1.6). The absolute percentage difference between adolescents’ and adults’ attendance was 3% at HCHC and 2% at CMJAH. Antenatal attendance in women above 30 years in CMJAH was higher than all other age groups in the other two facilities (94%, 6313/6700).

In HCHC only 95% of HIV-positive women had attended ANC (1803/1897), compared with 99% of negative women (3220/3270). HIV prevalence was similar in those who had or had not attended ANC in the other sites. Lastly, the proportion of women at CMJAH who had attended ANC rose step-wise with an increase in gravidity in the worst case, but not in the other two scenarios.

### Associations between antenatal attendance and HIV testing, delivery mode and birth outcomes

Overall, in the worst case scenario, HIV testing rates were 37% in women who had not attended ANC (1308/3528), compared with 93% of ANC attenders (25,756/27,651). Moreover, when separated by site, HIV testing rates among women not attending ANC varied considerably: from 8% at HCHC (144/1730) to 59% at SRH (268/452) to 67% at CMJAH (896/1346). In the missing-excluded scenario, the corresponding figures for SRH and CMJAH are 49% (1895/3859) and 99% (25,756/26,120). The respective proportions were 52% (2151/4115) and 99% (26,700/27,064) in the best case scenario.

In univariate analysis, rates of Caesarean section were considerably lower in women who had not attended ANC (13%, 422/3360) than those who had (40%, 10,866/27,344). Multivariate analysis showed similar findings and was consistent across all three scenarios (Table 2; Additional file 3: Table S3 and Additional file 4: Table S4).

No association was detected between ANC attendance and preterm birth in univariate analysis. However, in bivariate analysis stratified by facility, in each site the proportion of births that were preterm was higher in women who had not attended ANC than in those who had. This finding persisted across all scenarios. In multivariate analysis of the worst case scenario, non-attenders were 1.6 fold more likely to have a preterm birth than those who attended (aOR 95% CI = 1.4–1.8). The point estimates for this effect were 2.7 in the other two scenarios.

In the worst case scenario, ANC attendance was 89% in women who had a live birth (25,813/29,004), while only 81% of women with a stillbirth had attended clinic (431/535). In multivariate analysis of the worst case scenario, ANC non-attenders were 1.4 fold more likely to have had a stillbirth than their counterparts (aOR 95% CI = 1.1–1.9), figures similar to the other scenarios.

## Discussion

Within the inner-city of Johannesburg, only about 90% of women giving birth attended antenatal care – about 5% lower than the national average. Most non-attenders gave birth in the primary health care facility and substantially fewer had HIV testing or a Caesarean section. By not attending ANC, it is not possible to identify women who have high-risk pregnancies and refer them for closer monitoring during childbirth, or for an elective Caesarean section at a secondary or tertiary centre [24]. Thus, not surprisingly, not attending antenatal care was associated with a considerably higher risk for preterm delivery and stillbirths in this population, consistent with previous reports [4, 25–28]. Similarly, the high levels of maternal mortality in women not attending ANC in the country can likely be ascribed to the women not having attended the appropriate level of care during pregnancy and childbirth, and not having received interventions during pregnancy such antiretroviral treatment [13, 14]. In countries with high HIV prevalence, such as those in eastern and southern Africa, HIV is a leading cause of death among women during pregnancy and the postpartum period [29, 30].

Importantly, the study findings were broadly consistent across the sensitivity analyses done to test the effects of different assumptions about the missing data. Effects sizes in all four multivariate models were similar or even larger than the worst case scenario. The estimates of ANC attendance in the missing-excluded and best case scenario were, however, implausible in many instances (e.g. that 99% of women at SRH had attended ANC).

Despite recommendations for HIV testing in labour or shortly after childbirth in women with an unknown HIV status [31], HIV testing levels were low among women who had not attended ANC. This is true of all levels of care, but most especially at the primary care facility, where only about 10% of non-attenders had an HIV test. Non-attenders should be considered a very high risk group and prioritised for HIV testing around childbirth (HIV positivity rate was two thirds in non-attenders who had an HIV test). Independent of ANC attendance, women who did not have HIV testing had low rates of Caesarean section and markedly poorer birth outcomes than other women. This suggests that women not accessing HIV testing require considerable focused attention, as lack of HIV testing may signal that they have poor access to a range of interventions.

Being an adolescent or a woman living with HIV at the HCHC site lowered the chances of attending antenatal care during pregnancy. Although reasons for poor access were not assessed, likely factors include stigma (real or perceived), and concomitant disrespect and abuse meted out by health workers [32–34]. The especially low levels of attendance at HCHC, the Hillbrow site, where most residents are foreign nationals [35], suggests that xenophobia within the health system might be deterring women who are not South African from seeking health care [36, 37]. The Hillbrow site had the lowest levels of ANC attendance and HIV testing, and the highest HIV prevalence, and clearly serves a highly vulnerable and marginalised population who face many obstacles to service attendance.

Having attended ANC, regardless of number of visits, has been used for decades as an important measure of access to maternal health services [38–40]. The number of visits made to an antenatal clinic is, however, also a key measure of access, and was unfortunately not collected within birth registers in the study sites. The indicator ‘proportion of women attending four or more visits’ is one of the four indicators used to measure the target 3.8 of SDG 3, Universal Health Coverage of reproductive, maternal, newborn and child health [41, 42]. A multi-country trial led by WHO found that a minimum of four antenatal visits are required to adequately monitor a women’s health during pregnancy, and to complete screening and diagnostic procedures [43]. Additional visits may be necessary for HIV-positive women who initiate antiretroviral therapy during pregnancy and require adherence support and drug toxicity monitoring, for example [2, 19]. Globally, however, only an estimated 53% of women attend four visits and this figure is even lower in most LMICs (36%) [44, 45]. In South Africa, in a population-based survey, of those who attend ANC, 87% had four or more visits [7]. These figures, however, vary considerably between population groups, ranging from 81% in socio-economic quartile I to 97% in quartile IV, and were especially low in those aged below 19 (79%).

### Strategies to improve antenatal attendance

Clearly, in this setting, interventions to raise ANC attendance are a priority and critical to reducing maternal mortality, stillbirths and paediatric HIV, and to securing access to ART for HIV-infected women [12]. In the country as a whole, failure to address deficiencies in access to ANC, will substantially constrain the ability to further improve national maternal and child health outcomes.

Research identifying the specific demand-side barriers to access in this setting, especially within groups such as adolescents, HIV-positive women and foreigners, could provide actionable information. In other settings, these factors include lack of women’s empowerment and supportive partners [46], perceived poor quality of services [47], having an unintended pregnancy and low socio-economic status [48–50]. Demand for antenatal services could be raised through community involvement and activities to raise awareness around these services, including through mass media campaigns [17, 51, 52]. Further strengthening the antenatal care component of the government led MomConnect mHealth intervention might make an important contribution to such initiatives and to raising the number of ANC visits attended [53, 54]. Specific messages on MomConnect, or even a separate mHealth service, could be targeted at immigrants and younger poorer women. Cash transfers have been successfully applied in many settings to incentivise attendance at maternal health services [55]. Currently a cash transfer is given to women in South Africa once a child is born until they are 18 years. Beginning the transfer during pregnancy, as is done in a host of countries, (and not only restricting it to South African women) could raise demand for care (presentation of a ANC patient-held card could be required for enrolment in the scheme, for example) [56].

Supply side actions that have been found to raise attendance include improvements in service quality [57, 58]. More broadly, government should promote adolescent-friendly services and perhaps reserve specific time slots at the clinic for this group [51, 59]. Both within health services and the country as a whole, much more needs to be done to counter the high levels of xenophobia that mark the country and hinder access of migrants to health care [60]. Strategies to achieve this could include training and sensitisation of health workers, monitoring of health workers’ interactions with migrants and strengthening the coordination mechanisms between the multi-sectoral partners who work in this field [61]. Birth registers in areas with high numbers of foreign nationals could include measures of migrant status, such as country of origin and length of time resident in the country, in order to track outcomes of these women and identify any particularly vulnerable sub-groups to be targeted.

### Study strengths and limitations

Deficiencies in data quality, especially the gaps in data on ANC attendance at SRH and CMJAH, hinder the ability to interpret the findings. These deficiencies may partly be explained by the data having been collected as part of routine services, where staff have numerous competing priorities. However, regardless of what assumptions were made about the missing data, the direction and size of effect were generally consistent, suggesting that study findings may be robust despite this potential bias. Also, noting these deficiencies in data and the usefulness of the data collected is a key step towards improving data quality in the facilities and other parts of South Africa. Also of note, levels of attendance measured through health service data can differ considerably from coverage measured using population–level data [41]. However, where utilisation of skilled birth attendance is high, health service data, such as used in this study, may approximate antenatal coverage measured in a survey [62]. In Gauteng province, 99% of women deliver with a skilled birth attendant [7], suggesting that our measures of ANC attendance reflect that of population-level coverage.

Number of visits was not recorded in the birth registers, and thus we were unable to differentiate between those who had and had not completed the recommended four visits. Even attendance fewer times than the recommended number offers considerable benefits: the use of point-of-care HIV and syphilis testing, and of same-day initiation of antiretroviral treatment means that women attending even one visit can still access several important services.

The data collection periods differed between facilities. This could have biased the study findings as differences observed in attendance rates between the tertiary facility and other levels may be due to systematic improvements or reductions in ANC utilisation across the facilities, rather than due to the differences between levels of care. Also, patterns of patient referral may have shifted during that period and it is even possible that some women gave birth at the primary or secondary level facility in 2008–2009 and then later at the tertiary centre in 2011. It is likely, however, that the background population living in the study area remained relatively constant across the two periods. Moreover, overall, despite the data gaps and quality concerns, this was the only available data that provides information on this important topic. The use of disaggregated data from this large database is a major strength of the study, allowing us to identify differential access to services and variations in birth outcomes by sub-populations, who can then be targeted.

## Conclusion

Our study of birth registers in inner-city Johannesburg documented ANC attendance rates that are considerably lower than the country average, especially in the primary care clinic. Women who did not attend antenatal care during pregnancy had considerably poorer uptake of HIV testing, access to Caesarean sections and birth outcomes. Adolescents and HIV-positive women in primary care had lower attendance rates, which is particularly concerning as these groups are already vulnerable to a range of adverse health outcomes.

Our study demonstrated the importance of strengthening the antenatal component of MomConnect, for example, and of innovations such as cash transfers aimed at increasing ANC attendance. This is particularly critical for vulnerable groups such as HIV positive women, adolescents and immigrants. Maternal HIV testing around childbirth for women regardless of ANC attendance should be prioritised and strengthened. Although this has long been emphasized within national guidelines, it has not yet been fully actualised [63, 64]. Moreover, as a component of health systems strengthening within each sub-district, health promotion activities and community outreach related to maternal heath should be systematically assessed and reinforced to respond to facility-level data, such as that presented here [17, 65]. Finally, including measures of migration status in birth registers would enable ANC attendance to be disaggregated by migration status in future studies, potentially providing useful insights to guide local-level responses [37].

## Additional files

Additional file 1: Table S1. Proportion of women attending antenatal care in each facility, by maternal characteristics and birth outcomes in the missing excluded scenario (women with unknown ANC attendance status are excluded). (DOCX 22 kb)

Additional file 2: Table S2. Proportion of women attending antenatal care in each facility, by maternal characteristic and birth outcomes in best case scenario (women with unknown attendance classified as having attended ANC). (DOCX 22 kb)

Additional file 3: Table S3. Associations between antenatal attendance, and access to other services and birth outcomes in the missing excluded scenario (women with unknown ANC attendance status are excluded). (DOCX 22 kb)

Additional file 4: Table S4. Associations between antenatal attendance, and access to other services and birth outcomes in best case scenario (women with unknown attendance classified as having attended ANC). (DOCX 22 kb)

## Abbreviations

ANC: Antenatal care
ART: Antiretroviral treatment
CMJAH: Charlotte Maxeke Johannesburg Academic Hospital
HCHC: Hillbrow Community Health Centre
LMIC: Low- and Middle-Income Countries
PMTCT: Prevention of Mother-to-Child Transmission
SDG: Sustainable Development Goals
SRH: South Rand Hospital
