# Supplementary material for: Attendance at antenatal clinics in inner-city Johannesburg, South Africa and its associations with birth outcomes: analysis of data from birth registers at three facilities
Source: BMC Public Health. 2017 Jul 4;17(Suppl 3):443. doi: 10.1186/s12889-017-4347-z (PMC5498856; doi:10.1186/s12889-017-4347-z)
Supplement: Supplementary file 1 — Proportion of women attending antenatal care in each facility, by maternal characteristics and birth outcomes in the missing excluded scenario (women with unknown ANC attendance status are excluded). (DOCX 22 kb) [file 12889_2017_4347_MOESM1_ESM.docx]

**Table S1: Proportion of women attending antenatal care in each facility, by maternal characteristics and birth outcomes in the missing excluded scenario (women with unknown ANC attendance status are excluded)**

| **Variable** | **Primary care clinic** % attended (n/N) | ***P*** | **Secondary level hospital** % attended (n/N) | ***P*** | **Tertiary hospital**  % attended (n/N) | ***P*** |
| --- | --- | --- | --- | --- | --- | --- |
| **ANC attendance for facility** | 77.1 (5813/7543) |  | 99.1 (3661/4113) |  | 97.0 (18,177/19,523) |  |
| **Maternal age** (years)  10-16  17-19  20-24  25-29  30-34  ≥35 | 75.0 (66/88)  74.2 (542/731)  75.9 (2197/2893)  79.0 (1797/2274)  78.0 (844/1082)  77.6 (367/473) | 0.05 | - | - | 94.6 (229/242)  95.1 (1114/1172)  96.5 (4726/4899)  97.3 (5674/5831)  97.7 (3814/2903)  97.1 (2620/2698) | <0.001^$^ |
| **Parity**^  1  2-4  ≥5 | - | - | - | - | 97.1 (6046/6229)  96.8 (4642/4796)  94.4 (119/126) | 0.194^$^ |
| **Gravidity**^  1  2-4  ≥5 | - | - | - | - | 96.9 (5252/5420)  97.0 (11,761/12,127)  97.2 (1040/1070) | 0.869^$^ |
| **Had HIV test**  Yes  No | 97.2 (5023/5166)  33.3 (790/2375) | <0.001 | 99.7 (3353/3363)  93.3 (308/330) | <0.001 | 98.8 (17,380/17,591)  69.1 (797/1154) | <0.001 |
| **HIV status***  Positive  Negative | 95.1 (1803/1896)  98.5 (3220/3270) | <0.001 | 99.4 (798/803)  99.8 (2555/2560) | 0.05 | 98.5 (5180/5258)  98.9 (12,200/12,333) | 0.02 |
| **Delivery mode**  Vaginal delivery  Caesarean section | 77.1 (5813/7541)  NA | - | 98.9 (2741/3129) 100.0 (920/920) | 0.001 | 95.8 (7924/8270)  98.1 (9946/10,138) | <0.001 |
| **Infant sex**  Female  Male | 77.7 (2976/3833)  76.6 (2824/3686) | 0.28 | 99.3 (1795/1808)  99.0 (1864/1883) | 0.34 | 97.2 (8115/8636)  96.9 (8434/9017) | 0.38 |
| **Gestation at birth**  Preterm  Term  Post-term | 57.7 (15/26)  77.2 (5798/7515)  - | 0.02 | 90.5 (38/42)  99.2 (3616//3644)  100.0 (7/7) | <0.001 | 92.8 (4551/4903)  98.6 (12,318/12,498)  98.9 (866/876) | <0.001 |
| **Infant status at birth**  Alive  Stillbirth^ | 77.2 (5799/7508) 40.6 (13/32) | <0.001 | 99.2 (3647/3677) 92.9 (13/14) | 0.01 | 97.3 (16,367/16,821) 89.0 (405/455) | <0.001 |

NA=Not applicable as caesarean sections not done at HCHC. –Data not collected in labour ward registers. ^$^Chi-square test for trend. *HIV status among women with a known status. At HCHC, no births were considered post-term. Missing excluded: women known to have attended ANC, among those whose ANC attendance status was known ^Includes death shortly after birth
