# Supplementary material for: Attendance at antenatal clinics in inner-city Johannesburg, South Africa and its associations with birth outcomes: analysis of data from birth registers at three facilities
Source: BMC Public Health. 2017 Jul 4;17(Suppl 3):443. doi: 10.1186/s12889-017-4347-z (PMC5498856; doi:10.1186/s12889-017-4347-z)
Supplement: Supplementary file 3 — Associations between antenatal attendance, and access to other services and birth outcomes in the missing excluded scenario (women with unknown ANC attendance status are excluded). (DOCX 22 kb) [file 12889_2017_4347_MOESM3_ESM.docx]

**Table S3: Associations between antenatal attendance, and access to other services and birth outcomes in the missing excluded scenario (women with unknown ANC attendance status are excluded)**

| **Variable** | **Univariate odds ratio** | ***P*** | **Multivariate odds ratio** | ***P*** |
| --- | --- | --- | --- | --- |
| **Had an HIV test** | | | | |
| **Attended ANC**  Yes  No | 1.0  0.014 (0.012-0.015) | <0.001 | 1.0  0.020 (0.017-0.022) | <0.001 |
| **Site**  Primary level  Secondary level  Tertiary level | 1.0  3.38 (3.04-3.76)  6.74 (6.25-7.27) | <0.001  <0.001 | 1.0  1.8 (1.6-2.1)  3.8 (3.5-4.2) | <0.001  <0.001 |
| **Had a Caesarean section** | | | | |
| **Attended ANC**  Yes  No | 1.0  0.5 (0.4-0.6) | <0.001 | 1.0  0.5 (0.4-0.6) | <0.001 |
| **Site**  Secondary level  Tertiary level | 1.0  3.75 (3.47-4.05) | <0.001 | 1.0  3.5 (3.2-3.8) | <0.001 |
| **HIV status**  Negative  Positive  Unknown | 1.0  1.06 (1.00-1.13)  0.50 (0.45-0.56) | 0.04  <0.001 | 1.0  1.0 (0.9-1.1)  0.6 (0.5-0.7) | 0.833  <0.001 |
| **Gestation at childbirth**  Term or post-term  Preterm | 1.0  1.46 (1.37-1.56) | <0.001 | 1.0  1.24 (1.15-1.32) | <0.001 |
| **Preterm birth** | | | | |
| **Attended ANC**  Yes  No | 1.0  0.9 (0.8-1.0) | 0.23 | 1.0  2.7 (2.2-3.3) | <0.001 |
| **Site**  Primary level  Secondary level  Tertiary level | 1.0  3.34 (2.07-5.40)  106.6 (72.5-156.9) | <0.001  <0.001 | 1.0  8.0 (4.8-13.2)  276.3 (184.2-414.5) | <0.001  <0.001 |
| **Infant sex**  Male  Female | 1.0  0.98 (0.92-1.05) | 0.55 | - | - |
| **HIV status**  Negative  Positive  Unknown | 1.0  1.25 (1.16-1.33)  1.13 (1.03-1.24) | <0.001  <0.001 | 1.0  1.3 (1.2-1.4)  3.6 (3.1-4.1) | <0.001  <0.001 |
| **Stillbirth**^ | | | | |
| **Attended ANC**  Yes  No | 1.0  1.9 (1.5-2.5) | <0.001 | 1.0  1.6 (1.2-2.3) | 0.006 |
| **Site**  Primary level  Secondary level  Tertiary level | 1.0  0.92 (0.50-1.67)  6.57 (4.59-9.40) | 0.78  <0.001 | 1.0  2.1 (1.1-4.0)  17.9 (11.9-26.7) | 0.03  <0.001 |
| **HIV status**  Negative  Positive  Unknown | 1.0  1.12 (0.90-1.38)  2.38 (1.93-2.93) | 0.31  <0.001 | 1.0  1.1 (0.9-1.4)  4.1 (3.1-5.4) | 0.46  <0.001 |
| **Delivery mode**  Vaginal delivery  Caesarean section | 1.0  0.87 (0.72-1.05) | 0.14 | 1.0  0.5 (0.4-0.6) | <0.001 |
| **Infant sex**  Male  Female | 1.0  0.77 (0.65-0.92) | 0.004 | 1.0  0.7 (0.6-0.9) | 0.002 |

Caesarean sections not done at HCHC. No births considered post-term. Missing excluded: women known to have attended ANC, among those whose ANC attendance status was known ^Includes death shortly after birth
